# Human Cord Blood Endothelial Progenitor Cells and Pregnancy Complications (Preeclampsia, Gestational Diabetes Mellitus, and Fetal Growth Restriction)

**DOI:** 10.3390/ijms25084444

**Published:** 2024-04-18

**Authors:** Ja-Young Kwon, Yong-Sun Maeng

**Affiliations:** 1 Department of Obstetrics and Gynecology, Institute of Women’s Life Medical Science, Yonsei University Health System, Seoul 03722, Republic of Korea; jaykwon@yuhs.ac; 2 Department of Obstetrics and Gynecology, Yonsei University College of Medicine, 250 Seongsanno, Seodaemun-gu, Seoul 03722, Republic of Korea

**Keywords:** endothelial progenitor cells, preeclampsia, gestational diabetes mellitus, fetal growth restriction, angiogenesis

## Abstract

Hemangioblasts give rise to endothelial progenitor cells (EPCs), which also express the cell surface markers CD133 and c-kit. They may differentiate into the outgrowth endothelial cells (OECs) that control neovascularization in the developing embryo. According to numerous studies, reduced levels of EPCs in circulation have been linked to human cardiovascular disorders. Furthermore, preeclampsia and senescence have been linked to levels of EPCs produced from cord blood. Uncertainties surround how preeclampsia affects the way EPCs function. It is reasonable to speculate that preeclampsia may have an impact on the function of fetal EPCs during the in utero period; however, the present literature suggests that maternal vasculopathies, including preeclampsia, damage fetal circulation. Additionally, the differentiation potential and general activity of EPCs may serve as an indicator of the health of the fetal vascular system as they promote neovascularization and repair during pregnancy. Thus, the purpose of this review is to compare—through the assessment of their quantity, differentiation potency, angiogenic activity, and senescence—the angiogenic function of fetal EPCs obtained from cord blood for normal and pregnancy problems (preeclampsia, gestational diabetes mellitus, and fetal growth restriction). This will shed light on the relationship between the angiogenic function of fetal EPCs and pregnancy complications, which could have an effect on the management of long-term health issues like metabolic and cardiovascular disorders in offspring with abnormal vasculature development.

## 1. Introduction

A new paradigm for endothelial regeneration involving angiogenesis and vasculogenesis was established in 1997 with the identification of endothelial progenitor cells (EPCs) by Asahara et al. [1]. According to reports, endothelial progenitor cells (EPCs) are essential for the development of new blood vessels and the restoration of endothelial dysfunction [2,3]. EPCs are mostly found in the stem cells of bone marrow, while some are also present in the peripheral blood and cord blood [4,5]. EPCs are mobilized from bone marrow cells into the blood circulation in response to chemokine stimulation following a peripheral blood vessel injury or ischemic stroke. After injury, ischemia, or hypoxia, EPCs deposit in the endothelium to aid in tissue healing, as seen in both *human* and animal models [6,7,8]. Research has revealed the involvement of EPC in the processes of postnatal vasculogenesis, wound healing, myocardial infarction, and limb ischemia [9,10]. Growth factors such as vascular endothelial growth factor (VEGF)/KDR, granulocyte colony-stimulating factor (G-CSF), insulin-like growth factor 2 (IGF2), stromal cell-derived factor-1 (SDF1), hepatocyte growth factor, soluble intercellular adhesion molecule, interleukin-6 (IL-6), and endothelial nitric oxide synthase (eNOS) mediate EPC mobilization and differentiation in ischemic regions [11,12,13,14].

A crucial finding is that the quantity and function of endothelial progenitor cells (EPCs) are reduced as a result of senescence in conditions involving endothelial dysfunction, such as diabetes mellitus and cardiovascular disease [15,16,17,18], where the EPC count is a reliable indicator of future cardiovascular events [19]. Accelerated aging of these cells has also been linked to decreased EPC activity and number [20,21,22]. These data led to the hypothesis that diseases associated with endothelial dysfunction might be caused by the reduction of circulating EPCs.

Preeclampsia (PE) and intrauterine growth restriction in particular are regarded to be pathogenesis-related conditions that are influenced by aberrant vasculature and impaired endothelial function on both the maternal and fetal sides. The etiology of abnormal vessel development could perhaps result from decreased EPC function or bioavailability. Consequently, these pregnancy problems may be a result of impaired EPC function.

Pregnancies affected by preeclampsia or fetal growth restriction have been found in multiple studies to have a decreased number and abnormal function of cord blood-derived EPCs [23,24,25].

When the fetus’s growth-constrained, PE is linked to poor chorionic villous development and poor fetoplacental angiogenesis [26]. Significant decreases in cord blood EPCs and cord plasma free VEGF, which is known to be involved in EPC mobilization, were observed in severe preeclampsia [27]. Furthermore, in comparison with the normally derived EPCs, the preeclampsia-derived CD133+/C-kit+/Lin− EPCs (CKL− EPC) showed reduced out-growing endothelial cell (OEC) migration, adhesion, and tube formation activities, as well as delayed differentiation times and a significant decrease in the number of generated OEC colonies [23].

Maternal hyperglycemia caused by glucose intolerance that initially manifests during pregnancy is known as gestational diabetes mellitus (GDM). Hyperglycemia inhibits the clonogenic growth of cord blood endothelial colony-forming cells (ECFCs) by causing premature senescence [28]. Additionally, ECFCs exposed to a diabetic environment during pregnancy display lower proliferation and premature senescence [29,30].

One pregnancy condition that is clinically relevant is fetal growth restriction (FGR). A birth weight that is at or below the third centile for gestational age and abnormal dopplers are commonly used to determine FGR [31]. When compared with the normal control group, the fetal EPC numbers in the FGR group were considerably lower. The FGR group had a longer EPC differentiation period and significantly fewer OEC colonies [32,33].

These pregnancy diseases are linked to both maternal and perinatal morbidity as well as poor long-term results for the offspring, making them one of the major concerns for fetal development and the health of the offspring. Numerous research studies have demonstrated that children exposed to prenatal disorders are more vulnerable to long-term conditions such as obesity, metabolic syndrome, type 2 diabetes, and hypertension, all of which are subtypes of cardiovascular disease (CVD) [23,27,34,35,36,37,38].

In this review, we highlighted the role that fetal EPCs may play in the increased life-long cardiovascular risk that is associated with preeclampsia (PE), gestational diabetes (GDM), and fetal growth restriction (FGR), as well as their number, senescence, differentiation, and angiogenic function in the pathophysiology of these conditions.

## 2. Preeclampsia

### 2.1. Number of EPC in Preeclampsia

About 5–8% of pregnancies are complicated by preeclampsia (PE), a pregnancy disease with unclear etiology that greatly increases maternal and neonatal morbidity and mortality [39]. When the fetus is growth-constrained, PE is linked to poor chorionic villous development and fetoplacental angiogenesis [26,40]. Normal fetal growth is associated with increased branching angiogenesis and hyperramified capillary loops with uneven and narrow lumina [41,42]. It is appropriate to speculate that disturbances in EPCs may be implicated in this vasculopathy given their involvement in vascular homeostasis.

Recent research by Fadini et al. [43] revealed a decreased number of circulating EPCs in patients with chronic hypoxia with severe lung disease, and the reduction was related to impaired alveolo-arterial diffusion. Studies by Vasa et al. [15] and Hill et al. [44] also demonstrated a negative correlation between circulating EPCs and coronary artery disease risk factors. These data led to the hypothesis that diseases associated with endothelial dysfunction might be caused by the reduction of circulating EPCs. Additionally, a number of animal studies and modest clinical trials have shown the therapeutic efficacy of EPC transplantation in tissue ischemia, indicating a potential role for EPCs in the management of vascular disease [45,46,47,48].

We examined the circulating EPC number of umbilical cord blood in the normal and preeclamptic groups in earlier research. Table 1 displays the features of the patient. There were no discernible variations between the two groups’ gestational ages at delivery or patient ages. On the other hand, the severe preeclampsia group had a much greater rate of small for gestational age (SGA), lower birth weight and a significantly higher maternal systolic blood pressure. When compared with the normal group, the preeclampsia group’s circulating EPC number in the umbilical cord blood significantly decreased. [27,49,50]. Concurrently, the same group’s umbilical cord plasma showed a decline in the free VEGF level. Nevertheless, the rise in sVEGFR-1 did not attain statistical significance [27]. Additionally, Xia et al. demonstrated that, in the preeclampsia group as opposed to the normal group, there is a significant decrease in the placental/fetal circulating EPC numbers (median, 200; range, 100–440 cells/mL vs. 390; 270–440 cells/mL, *p* < 0.001) [51]. Following the in vitro culture, the preeclampsia group’s EPC counts likewise declined (19.5; 5.0–32.0 vs. 39.5; 31.2–52.0 EPC/ × 200 field; *p* < 0.001). There was an inverse correlation between the level of soluble fms-like tyrosine kinase 1 (sFlt-1) in cord blood and both circulating and cultured EPC [51].

These results imply that in cases of severe preeclampsia, ineffective EPC mobilization from the bone marrow due to a reduction in free VEGF as a consequence of increased binding to excess sVEGFR-1 is the cause of the decreased EPCs in the umbilical cord blood. We were unable to show a correlation between decreased cord plasma free VEGF and increased cord plasma sVEGFR-1 despite the fact that the EPC count was lower in the cord blood from preeclamptic pregnancy.

### 2.2. Senescence of EPC in Preeclampsia

Senescent cell burden in adipose, skeletal muscle, kidney, and skin tissues is low in young people but rises with age [52,53,54]. Specifically, among the many diseases linked to elevated senescent cell load are parts of the metabolic syndrome, such as diabetes, atherosclerosis, hypertension, and abdominal obesity [55,56,57].

Maternal vascular/endothelial malfunction and placental deficient angiogenesis are the hallmarks of preeclampsia [58,59]. Numerous investigations revealed that vascular endothelial dysfunction is significantly influenced by cellular senescence [60,61,62,63].

Senescence of EPC in maternal circulation associated with preeclampsia has been recently observed. Patients (maternal age: 32.4 ± 1.4) with preeclampsia had a considerably higher rate of cellular senescence (33.9%) than controls (maternal age: 31.8 ± 2.1) (22.9%; *p* < 0.05) [64]. Furthermore, patients diagnosed with preeclampsia were separated into two subgroups: those with C-reactive protein (CRP) levels less than or equal to 0.1 mg/dL (*n* = 4) and those with CRP levels greater than or equal to 0.1 mg/dL (*n* = 4). Cellular impairment of EPCs was linked to serum levels of C-reactive protein (CRP), a sign for systemic inflammation. It is interesting to note that the CRP-positive group had higher median values for cellular senescence than the CRP-negative group did; however this difference did not reach statistical significance (43.5% and 33.3%, respectively; *p* = 0.12) [64].

Additionally, the in vitro features of EPC cellular aging were evaluated using the senescence-associated β-galactosidase (SA-β-gal) activity assay for fetal EPCs obtained from women with preeclampsia and normal pregnancies.

Compared with normal pregnant women (32.4%; range, 25.2–39.6%; *p* < 0.001), patients with PE had a considerably higher number of SA-β-gal-positive cells (55.8%; range, 49.5–62.1%) [49]. These results show that endothelial dysfunction may be linked to the cellular aging of fetal EPCs in preeclamptic individuals.

### 2.3. Differentiation Activity and Angiogenic Function of EPC in Preeclampsia

In addition to the immediate implications on the fetus, such as preterm or fetal growth restriction, maternal preeclampsia may also result in potential problems with the fetus’s vascular health due to effects sustained in utero. The findings of several additional studies, which have previously documented a higher prevalence of adult-onset metabolic and cardiovascular disorders, including hypertension, cardiovascular disorders, cerebrovascular disorders, impaired neural development, type 2 diabetes, metabolic syndrome, and hypercortisolism in people born to preeclamptic women, provide support to this [65,66,67,68,69].

Because EPCs are endothelial cell progenitors and can therefore regulate angiogenesis, neovascularization, and vasculogenesis postnatally [46,70], it is likely that a variety of vascular disorders are caused by their malfunction. Indeed, previous research has demonstrated a decrease in the quantity of circulating EPCs in vascular and metabolic conditions, including metabolic syndrome, coronary artery disease, diabetic vasculopathy, atherosclerosis, and systemic lupus erythematosus [71,72,73,74,75]. It has been demonstrated that preeclampsia, which can be defined as a hypertensive disease, is linked to a lower level of circulating EPCs in peripheral blood vessels [76].

Because cord blood is a part of the fetal circulation, the angiogenic property of cord blood EPCs most likely resembles the fetus’s total angiogenic potential. The findings of an earlier investigation, which demonstrated that OECs differentiated from cord blood EPCs and had angiogenic potential [77], are consistent with this. It is known that there are fewer cord blood-derived EPCs in preeclamptic pregnancies and that the few EPCs that are found are primarily senescent, despite the fact that little research has been conducted to date to examine the function of fetal EPCs exposed to preeclampsia [27,49,78,79].

Proliferation, migration, and vasculogenesis were all markedly compromised in the AC133+/KDR+ EPC from the cord blood of preeclamptic individuals [51]. Furthermore, compared with cord blood-derived CKL− EPCs from normal pregnancy, preeclampsia pregnancy-derived cord blood CKL− EPCs and the resulting differentiated OECs showed decreased differentiation capacity and angiogenic function, respectively [23]. Immature differentiation of OEC for angiogenesis is shown by diminished migration, adhesion, and tube formation function of preeclampsia-generated OECs in comparison with normal OECs. Moreover, exposure to normal serum conditions (17 days) did not restore the preeclampsia-derived CKL− EPCs’ reduced differentiation potency, indicating that their impaired function might be irreversible [23]. Conversely, CKL− EPCs produced from normal donors were able to differentiate even in the presence of preeclamptic serum (13 days) in culture media (vs. control serum: 8 days) [23]. Therefore, via influencing fetal vasculogenic/angiogenic function, the preeclamptic intrauterine environment appears to permanently alter pathways that significantly regulate and maintain fetal vascular health.

Environmental factors can induce permanent alterations in gene expression and/or protein function through a common process known as epigenetic modifications. A high frequency of transcriptionally competent yet changed genes, characterized by both active and repressive histone modifications linked to significant processes of differentiation and development, is a defining feature of stem and progenitor cells [80]. H3K4me3 and H3K9me3 levels were observed to be lower in the CKL− EPCs obtained from preeclampsia in the previous investigation [23]. Histone alterations have the potential to trigger pro-angiogenic (H3K4me3) and/or repressive (H3K9me3) signaling pathways, perhaps signifying a change in the proangiogenic/antiangiogenic balance in favor of anti-angiogenesis.

Collectively, these results demonstrated that the differentiation potency of cord blood EPCs produced from preeclampsia was irreversibly lower than that of EPCs derived from normal cases. Also, there was a notable reduction in the angiogenic potential of OECs that differentiated from EPCs produced from preeclampsia. To determine if the reduced function of preeclampsia-derived cord blood EPCs lasts after delivery and to clarify the mechanisms by which preeclampsia-induced effects on EPCs affect the exposed fetus’s long-term vascular health, more research is necessary.

## 3. Gestational Diabetes Mellitus (GDM)

### 3.1. Number of EPC in GDM

Maternal hyperglycemia caused by glucose intolerance that initially manifests during pregnancy is known as gestational diabetes mellitus or GDM. It has been steadily rising globally along with the increase in obesity among women of reproductive age and older mothers, complicating 5–31.5% of pregnancies [81,82]. Europe had the lowest prevalence of GDM (median 5.8%; range 1.8–22.3%), while the Middle East and North Africa had the highest overall, with a median estimate of 12.9% (range 8.4–24.5%). Southeast Asia, Western Pacific, South and Central America, Africa, and North America and the Caribbean followed with 11.7%, 11.2%, 8.9, and 7.0%, respectively, as the next highest prevalence regions. Different GDM diagnosis criteria were applied to the prevalence estimates, which varied greatly across the Western Pacific area, ranging from 4.5% in Japan to 25.1% in Singapore. The prevalence of GDM varied greatly throughout Europe as well; according to the WHO 1999 and the modified International Association of Diabetes and Pregnancy Study Groups criteria, Norway had the highest prevalence (median 22.3%; range 13.0–31.5%), while Ireland had the lowest prevalence (1.8%, according to the National Institute for Health and Care Excellence criteria) [82].

Offspring of women with gestational diabetes mellitus (GDM) are more likely to develop obesity, metabolic syndrome, and cardiovascular disease. A growing amount of research indicates that intrauterine hyperglycemia affects endothelial function in several fetal tissues and permanently changes genes’ epigenetic alterations.

Reduced number and impaired function of EPCs were shown in patients with endothelial dysfunction, and impaired EPC-mediated vascular repair allows for further progression of vascular disease. This is because the number of EPCs and their functional capacity determine the maintenance of endothelial homeostasis and de novo vessel formation [83,84,85,86]. Cord blood-derived EPCs, which are also referred to as fetal EPCs, can play a role in the development of the fetal vasculature and preservation of vascular integrity. As a result, dysfunction of these fetal EPCs may be a reflection of stem cell damage, which may lead to vascular dysfunction in the offspring later in life. Nevertheless, it is still unclear if fetal EPC dysfunction is a result of intrauterine hyperglycemia driven on by gestational diabetes mellitus (GDM) and what the underlying mechanisms involve.

EPCs have been demonstrated to be reduced in diabetes [16] and play an essential role in the neovascularization process that occurs following damage. When comparing the amount of maternal EPCs isolated from GDM with a normal pregnancy, Penno G et al. found that there was a substantial decrease in circulating EPCs in GDM [83]. Additionally, compared with non-diabetic patients, GDM patients had a statistically significant decrease in the percentage of circulating EPC from maternal blood (0.26% vs. 0.41%, respectively; *p* < 0.05) [87]. According to Gui J et al., there was a decrease in the number of progenitor cells called endothelial colony-forming cells (ECFCs) generated from umbilical cord blood in an intrauterine environment that was diabetic as compared with healthy pregnancies [88]. In GDM pregnancies, the average number of cord blood ECFC colonies appearing in culture was substantially lower (GDM: 2.94 ± 1.66, uncomplicated pregnancies: 5.83 ± 3.41, *p* = 0.04). In GDM pregnancies, the average days to first appearance of colonies appeared slightly later (GDM: 11.06 ± 4.14, *n* = 18; uncomplicated pregnancies: 9.95 ± 4.31), but not substantially later (*p* = 0.43). For ECFCs from GDM pregnancies, the population doubling period was much longer (GDM: 4.17 ± 1.23 days, uncomplicated pregnancies: 2.31 ± 0.43 days, *p* = 0.02). Colony count, colony appearance time, and population doubling times did not significantly correlate with any other patient variables [88].

Although there was a difference in umbilical cord EPCs between GDM patients and control patients, it was not statistically significant (1.76% vs. 1.46%, respectively) [87]. Furthermore, in our earlier research, the quantity of fetal EPCs produced from cord blood did not alter in GDM pregnancies. These results suggest that prenatal exposure to GDM does not affect the quantity of fetal EPCs, and more research is required.

### 3.2. Senescence of EPC in GDM

The function of EPCs may be reduced by premature senescence associated with gestational diabetes mellitus (GDM) [89,90]. In tissue stem cells, aging is a universal, progressive, and irreversible loss of function [91]. Furthermore, diabetes has been frequently linked to lower levels of circulating EPCs [92,93,94,95]. Endothelial progenitor cells (EPCs) in circulation are crucial for vascular regeneration. Nevertheless, it is still unclear how high hyperglycemia (HG) causes cord blood EPC aging. According to Wu et al. and Ingram et al., HG markedly raised the proportion of cord blood CD34+ EPC senescent cells [90] and cord blood endothelial colony-forming cells (ECFCs) that underwent premature senescence [29], respectively. Additionally, we examined the impact of GDM on fetal EPC senescence in our earlier research, and the results indicated a considerable rise in SA-β-gal positive CKL− EPCs. These results show that endothelial dysfunction may be linked to the cellular aging of fetal EPCs in GDM patients.

### 3.3. Differentiation and Angiogenic Function of EPC in GDM

Because GDM is linked to both maternal and perinatal morbidity as well as unfavorable long-term effects in offspring, it is one of the major issues for fetal development and offspring health. Numerous studies have revealed that children exposed to gestational diabetes mellitus (GDM) are more vulnerable to long-term conditions such obesity, metabolic syndrome, type 2 diabetes, and hypertension, all of which are subtypes of cardiovascular disease (CVD) [34,35,36,96,97].

Several investigations have indicated that pregnancies complicated by preeclampsia or fetal growth restriction were associated with a lower number and abnormal function of cord blood-derived EPCs [23,24]. Nevertheless, it is yet unclear if intrauterine hyperglycemia brought on by GDM influences fetal EPC development and angiogenic activity.

According to Gui J et al., compared with those from healthy pregnancies, the number and function of endothelial colony-forming cells (ECFCs) produced from umbilical cord blood were decreased in a diabetic intrauterine environment, including proliferation, migration, and tube formation [88,98]. They further showed that long-term cardiovascular disorders in the offspring of GDM pregnancies may be linked to lower sirtuin (SIRT) expression and activity in fetal ECFCs and *human* umbilical vein endothelial cells (HUVECs) from GDM pregnancies [99].

Based on prior research, we postulate that intrauterine exposure to gestational diabetes mellitus (GDM) induces endothelial dysfunction by modifying the function of fetal endothelial progenitor cells (EPCs) via epigenetic modifications in gene expression, eventually affecting the offspring’s subsequent developmental processes [30,87,100,101].

Growing data indicate that the development of diabetes-related vascular disease in progeny is influenced by epigenetic changes through DNA methylation in GDM [102,103,104,105,106]. Genomic stability and gene expression are controlled by DNA methylation. In our investigation, we discovered a negative correlation between hypomethylation and PCDH10 mRNA expression in OECs produced from GDM EPCs and OECs derived from N-EPCs subjected to high glucose levels. According to these findings, promoter hypomethylation is the main regulatory mechanism for fetal EPCs exposed to high glucose levels or GDM to express PCDH10 more than normal, which ultimately results in abnormal vascular health in the progeny. Furthermore, additional cohort studies are required to verify if these modifications continue to influence the offspring’s vascular health later in life. Also, additional research on angiogenesis-related genes like *ROBO1*, *IGFBP1*, and *PCDH7* may be attempted in regard to the gene expression study of OECs from GDM EPCs. The objective of our study was to explore the programming effects of GDM on fetal EPCs and clarify the underlying mechanisms that may be associated with unfavorable outcomes in progeny. We used a cell migration, adhesion, tube formation, and proliferation assay to measure the angiogenic activity of OECs that were differentiated from cord blood-derived EPCs in order to investigate at how GDM affected the fetal EPCs. When compared with normal EPCs, GDM EPCs showed comparable differentiation activity in terms of both number and time of differentiation to OECs [107]. However, GDM OECs demonstrated significantly lower migration capacity, fibronectin adherence, tube formation, and proliferative activity [107].

Fetal EPCs from normal pregnancies were grown in high glucose (30 mM) during differentiation into OECs in order to mimic an intrauterine hyperglycemic circumstance. These cultures were then compared with those cultured in normal glucose (5 mM). Similar to GDM EPCs and GDM OECs, OECs differentiated by exposure to high glucose (HG) had reduced capacity in cell migration, adhesion, tube formation, and proliferation compared with those under normal glucose (NG) conditions, even though there was no discernible difference in the differentiation from EPCs to OECs between NG and HG conditions [107].

In addition, we identified a number of genes linked to decreased fetal EPC function in GDM and discovered epigenetic modifications affecting gene expression to further comprehend the pathology of endothelial dysfunction in GDM-exposed offspring.

These results showed that, whereas fetal EPCs exposed to GDM can differentiate into OECs under normal circumstances, in utero exposure to GDM reduces the angiogenic potential of OECs, including migration, adhesion, tube formation, and proliferation. It makes sense to assume that an intrauterine hyperglycemic environment at crucial stages of fetal vascular development causes persistent damage to the offspring’s vascular system.

## 4. Number and Senescence of EPC in FGR

One pregnancy complication that is clinically relevant is fetal growth restriction (FGR). A birth weight that is at or below the third centile for gestational age and abnormal dopplers is commonly used to determine FGR. [31] The fact that 13% of instances do not exhibit postnatal catch-up growth is the reason for the growing interest in FGR [108]. Up to 5% of pregnancies may be affected by the common condition known as FGR, which has a significant morbidity and mortality rate. Low birth weight for gestation babies are more likely to have long-term developmental disorders as well as neonatal morbidity and mortality [37]. A significant risk of perinatal problems is often linked to FGR [109,110]. An increasing amount of research on animals and epidemiology has shown that the effects of FGR persist throughout adulthood [38]. The majority of FGR cases are idiopathic, meaning their cause is unclear, and treatment approaches are ineffective [111]. However, an important proportion of cases, most cases defined as fetal growth restriction, are related to placental insufficiency and dysfunction. Adult disorders like diabetes mellitus and cardiovascular disease have been the focus of EPC-related research up to now. In actuality, fetal blood contains a higher concentration of EPCs than adult blood does, and pathologic diseases related to endothelial cells, such as FGR and preeclampsia, provide a serious clinical problem during pregnancy.

In adulthood, an increasing amount of research indicates that endothelial dysfunction-related disorders such as cardiovascular disease [15], diabetes mellitus [16], and preeclamptic pregnancy [64] are associated with impairments in the number and function of EPCs. Similar to this, numerous studies have proposed a link between children born with FGR and the development of the metabolic syndrome in adulthood, which includes diabetes, cardiovascular disease, asthma, intellectual disabilities such as depression and schizophrenia, and a lower intelligence quotient. This link has been considered as “fetal programming” [112,113,114]. Therefore, if there was any impairment of the fetal EPCs in FGR, it may have affected future adult life as well as intrauterine fetal growth, even if it is unclear whether the depletion and enhanced aging of EPCs in these diseases are causes or outcomes.

The cord blood from the FGR group had a mean EPC count per 50 mL that was considerably lower (4.3 ± 1.0 × 10^4^/50 mL) than that of the normal group (6.6 ± 1.2 × 10^4^/50 mL), as demonstrated by Hwang et al. [32]. Furthermore, the FGR group’s 8.8 ± 1.5-day differentiation period from EPC to OEC was longer than the normal group’s 6.0 ± 1.2-day differentiation period (*p* < 0.001). The staining intensity of SA-β-gal-positive cells was relatively higher in the FGR group compared with the normal group (141.3 ± 13.6% vs. 100.0 ± 14.82%; *p* < 0.001), and the OEC colony number from 1 × 10^5^ fetal EPCs in each group was significantly decreased in the FGR group (7.9 ± 0.9 × 10^5^ EPC) compared with the normal group (11.8 ± 1.6 × 10^5^ EPC) (*p* < 0.001). Additionally, the FGR group’s telomerase activity was noticeably lower than that of the normal group (70.3 ± 6.5% vs. 100.0 ± 9.4%; *p* < 0.001).

In summary, circulating fetal EPCs in FGR are more senescent, have a lower differentiation capacity, and are fewer in number. These findings imply that the functional and numerical impairment of fetal EPCs in idiopathic FGR offers a likely explanation and could point to a practical intrauterine growth restriction treatment approach. Additionally, a number of studies found that during an FGR pregnancy, the concentrations of several known inflammatory, hypoxic, and antiangiogenic markers were higher in the cord blood [115,116]. These findings could help to explain why fetal EPCs in FGR pregnancies have lower numbers, decreased functional ability, and higher senescence.

## 5. Conclusions

**Figure 1:**
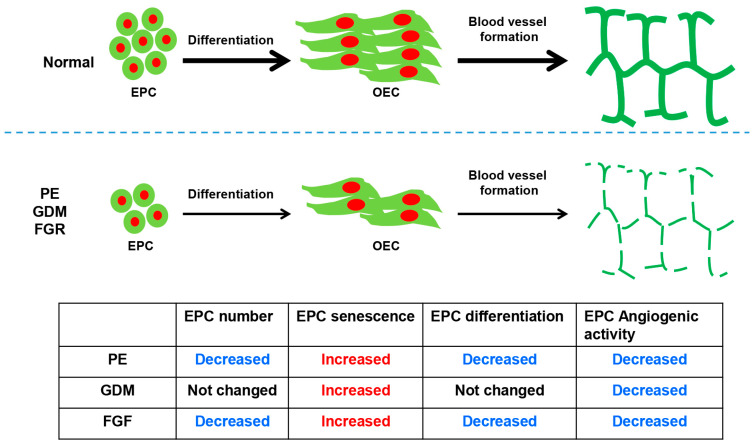
Proposed model describing the vessel formation of EPCs in the pathophysiology of preeclampsia (PE), gestational diabetes (GDM), fetal growth restriction (FGR), and normal pregnancy conditions. The fetal EPCs of pregnancy complications displayed decreased blood vessel formation activity.

In this review, we address the importance of fetal EPCs in the pathophysiology of fetal growth restriction (FGR), gestational diabetes (GDM), and preeclampsia (PE) (Figure 1). First, we proposed that preeclamptic patients had a considerably higher rate of fetal EPC senescence than controls did. Furthermore, in comparison with the normally derived EPCs, the preeclampsia-derived EPCs showed lower angiogenic activity, delayed differentiation periods, and a significant decrease in the number of produced OECs colonies. Second, we concluded that prenatal exposure to GDM reduces the angiogenic potential of EPCs and causes a marked increase in fetal EPC senescence in the GDM group. Nonetheless, there was no difference in the number of fetal EPCs obtained from cord blood or in the differentiation activity of EPCs during GDM pregnancy. Third, we postulated that there are less circulating fetal EPCs in FGR, and they have lower angiogenic activity and differentiation potential and are more senescent. As a result, this study offers a thorough explanation for the role that cord blood fetal EPC plays in pregnancy problems and suggests that EPC may play a role in the elevated lifetime cardiovascular risk that the offspring of pregnancy disorders may face. Therefore, it is suggested that restoration of EPC function through correction of genes related to the decline in angiogenic function of EPCs in woman with gestational disease can be a way to suppress the development of diseases caused by vascular dysfunction. In addition, we suggest that it can be used as a predictive diagnostic marker for the development of gestational vascular disease by discovering specific genes abnormally expressed in the EPCs of pregnant woman.

## Figures and Tables

**Table 1:** Patient characteristics.

	Normal Pregnancy (*n* = 30)	Preeclampsia (*n* = 30)	*p*-Value
Maternal age (years)	33.7 ± 5.3	32.75 ± 3.1	0.549
Gestational age at delivery (weeks)	38.65 ± 0.8	36.35 ± 2.1	0.386
Gravida	2.5 ± 1.4	1.55 ± 1.0	0.05
Pre-pregnancy body mass index (BMI) (kg/m^2^)	21.75 ± 4.7	22.1 ± 3.8	0.387
Blood pressure (mmHg)			
Systolic blood pressure	115.1 ±7	151.05 ±15	<0.015
Diastolic blood pressure	74.25 ±15	95.7 ±18	<0.015
Birth weight (g)	3301.0 ± 365	2303.1 ± 536	<0.001
Small gestational age (n)	0	21	0.03
Free VEGF, mean (SD)	22.66 (12.88)	12.84 (1.56)	0.04
sVEGFR-1, median (range)	1.11 (1.66)	1.25 (27.81)	0.24
EPC count (×10^6^/50 mL)	8.61	2.65	0.009

## Data Availability

The data that support the findings of this study are available from the corresponding author upon reasonable request.
